# Psychometric properties of the Shirom-Melamed Burnout Measure (SMBM) among adolescents: results from three cross-sectional studies

**DOI:** 10.1186/s12888-018-1841-5

**Published:** 2018-08-25

**Authors:** Markus Gerber, Flora Colledge, Manuel Mücke, René Schilling, Serge Brand, Sebastian Ludyga

**Affiliations:** Department of Sport, Exercise and Health, University of Basel, St. Jakobsturm, Birsstrasse 320B, 4052 Basel, Switzerland

**Keywords:** Burnout, Mental wellbeing, Measurement invariance, Stress, Validity

## Abstract

**Background:**

Burnout has long been understood as work-related physical, emotional, and cognitive exhaustion. However, burnout symptoms can also be found among younger people, including school-aged adolescents. While the Shirom-Melamed Burnout Measure (SMBM) is a widely applied instrument, its psychometric properties have not yet been investigated in adolescent populations. We therefore examined the psychometric properties of the SMBM in three independent samples of adolescents.

**Methods:**

In total, 249 high school students, 144 vocational students, and 257 adolescent elite athletes completed the SMBM, along with questionnaires related to perceived stress, depressive symptoms, and life satisfaction. Descriptive statistics, gender differences, and internal consistency, convergent/discriminant validity, and factorial validity (including measurement invariance across genders) were examined in each sample.

**Results:**

The SMBM had adequate internal consistency. Confirmatory factor analyses showed that both a first- and second-order model achieve good model fit. Moreover, evidence for sufficient convergent and discriminant validity was found. Finally, in two of the three samples, female adolescents reported higher SMBM scores.

**Conclusions:**

The SMBM has been widely used in international burnout research. However, this is the first study providing empirical evidence that the SMBM has acceptable psychometric properties and satisfactory convergent/discriminant and factorial validity among young people. The SMBM is a concise and economic tool to assess self-rated symptoms of burnout, and presents a valuable alternative to existing school burnout inventories. In particular, the SMBM can facilitate the investigation of the transition of young people from school to working life.

**Electronic supplementary material:**

The online version of this article (10.1186/s12888-018-1841-5) contains supplementary material, which is available to authorized users.

## Background

Burnout is a complex phenomenon with more than 40 years of empirical research [1]. Burnout was initially defined as a work-related syndrome that gradually develops when people are exposed to chronic emotional and interpersonal stress at work [2]. While researchers generally agree that burnout is a multidimensional construct [3–5], different conceptualizations of burnout have been proposed by different groups of researchers [6]. Drawing on Hobfoll’s Conservation of Resources (COR) theory, Melamed et al. [7] argued that the central characteristics of the burnout construct are *emotional exhaustion*, *physical fatigue*, and *cognitive weariness*.

Based on Shirom, Melamed et al.’s [8, 9] definition of burnout, researchers have shown that burnout has an impact on both physical and mental health outcomes: For instance, burnout was found to be a risk factor for increased total cholesterol, low-density lipoprotein cholesterol and triglyceride levels [7, 10–12], increased fasting glucose and risk of developing type 2 diabetes [13, 14], increased inflammatory markers [12, 15], increased leukocyte adhesiveness [16], increased diurnal cortisol levels [17], elevated cortisol response after awakening [18], increased risk of musculoskeletal pain [19], and a higher likelihood of fertility problems [20]. As regards psychological dimensions, data revealed that burnout symptoms constitute a risk for poor life satisfaction and quality of sleep [17, 21]. Similarly, significant relationships were observed between burnout and depression, with varying degrees of overlap [15, 22–24].

While the above findings underscore that burnout symptoms are a cause of concern from a public health perspective [25], most of the existing evidence is based on working adults and therefore cannot be generalized to young people. Nevertheless, several researchers have claimed that the concept of burnout is pertinent beyond the occupational context and also concerns student populations [26, 27]. More specifically, Salmela et al. [27] have argued that school-aged adolescents and university students – by attending classes, completing assignments, taking examinations, and acquiring a degree – also execute work. As a consequence, researchers have developed instruments specifically designed for young people. For instance, based on the Maslach Burnout Inventory (MBI) [4], Schaufeli et al. [26] have created a 15-item instrument for university students, which consists of three dimensions: *exhaustion*, *cynicism*, and *professional efficacy*. This instrument was well received by the scientific community, and has stimulated a large number of studies to examine the associations with academic performance and various health-related outcomes [28–34]. Some years later, Salmela-Aro and Näätänen [35] developed an instrument to assess burnout among school-aged children, the School Burnout Inventory (SBI). On the basis of the Bergen Burnout Indicator 15 (BBI-15) [36, 37], burnout was conceptualized as a three-dimensional construct consisting of the following subdomains: *exhaustion at school* (four items), *cynicism toward the meaning of school* (three items), and *sense of inadequacy at school* (three items). Evidence supported the validity and reliability of the SBI [27], and based on this tool, researchers were able to gain further insights into risk factors [38–44], time courses [42, 45], and health consequences associated with school burnout [46–48] among adolescents.

In summary, it can be concluded that research on burnout among school-aged adolescents has increased significantly in the last ten years. However, it is also obvious that the existing research has been dominated by the SBI. While this instrument has sound psychometric properties [27], the school-specific conceptualization of the construct makes it difficult to compare the SBI scores with those of adult populations, and complicates research focusing on the transition from school to working life. As highlighted by Walburg [49] in her review on adolescent school burnout, only a few alternative instruments have been applied in this age group. Nevertheless, some researchers have used the Shirom Melamed Burnout Measures (SMBM) [7, 16] as an alternative to assess burnout symptoms in adolescent students. In these studies, higher scores on the SMBM were associated with more depressive symptoms, more sleep problems, lower life satisfaction, and poorer quality of sleep [50]. Moreover, adolescents who accomplished recommended levels of physical activity reported lower SMBM scores [51], and the relationship between adolescents’ stress and burnout scores was moderated by their levels of mental toughness [52].

Although the SMBM seems to have satisfactory internal consistency among adolescents [50–52], the validity and reliability of the SMBM have not yet been examined systematically in young people. Compared to the SBI, the advantage of the SMBM is that the measure is rooted in Hobfoll’s Conservation of Resources theory [53], and thus has a clear theoretical background. Moreover, the items of the SMBM are context unspecific, and thus allow a comparison between varied samples of adolescents. Finally, the SMBM allows a comparison between burnout symptoms of adolescents and adults, is well suited to examining transitions from adolescence to adulthood, and provides a cut-off pointing towards clinically relevant levels of burnout [54]. Therefore, the purpose of the present study was to validate the SMBM in three samples of adolescents attending different types of public schools in Switzerland. We claim that these analyses are warranted because the SMBM has been widely used in (adult) burnout research during the last 25 years [24], and because it is time to find out whether this instrument is also suitable for younger people.

In the present article, six hypotheses will be tested: First, we expect that the SMBM will produce adequate internal consistency across all student samples. Thus, we expect inter-item correlations of ≥ .20, Cronbach’s alpha coefficients of ≥ .70, and item-total correlations of ≥ .30 [16, 55, 56]. Second, regarding factorial validity, we expect that adequate model fit will be found for a three-factor model [55, 56]. We also expect that adequate model fit will occur for a first- and second-order model [55]. More specifically, with reference to the standards defined by Comrey et al. [57] and based on previous findings [55, 56], we expect very good factor loadings (≥ .63) across all items on the corresponding factors. Third, we expected to find adequate convergent validity. That is, we hypothesize that the SMBM subscales and the SMBM overall index will be moderately to strongly (and positively) correlated with perceived stress [58–60] and the School Burnout Inventory (SBI) [51],^1^ while we expect a moderate (negative) relationship between the SMBM and adolescents’ life satisfaction [61]. Fourth, as an indication of discriminant validity, we assume that only moderate (positive) correlations will exist between the SMBM and self-reported depressive symptoms [24, 61]. Fifth, we expect that girls will score higher on the SMBM than boys [59, 61, 62]. Sixth, we expect the measurement model to be invariant across samples.

## Methods

### Sample 1: High school students

#### Participants and procedures

Sample 1 consisted of 249 students attending three high schools in the North-Western, German-speaking part of Switzerland (age: *M* = 16.09 years, *SD* = 1.00, 89 boys, 160 girls). From each school, five classes were randomly selected. Paper-and-pencil questionnaires were completed in a group setting during official class time (during the second month of the school year between September–October). Students were informed that participation was voluntary and that their responses would be treated confidentially. Inclusion criteria were: (a) informed written consent, (b) aged between 14 and 20 years, and (c) attending a selected class of one of the three high schools. None of the eligible participants had to be excluded. All participants provided written informed consent^2^. The study was approved by the local ethics committee.

#### Burnout

Burnout symptoms were assessed with the 14-item SMBM [16, 17]. The original SMBM consists of the three subscales labelled *physical fatigue* (six items: e.g., “I feel physically drained.” or “I feel fed-up.”), *cognitive weariness* (five items: e.g., “I feel I am not thinking clearly.” or “I have difficulty concentrating.”), and *emotional exhaustion* (three items: e.g., “I feel I am unable to be sensitive to the needs of coworkers and customers.” or “I feel I am not capable to being sympathetic to coworkers and customers.”). The wording of the three items assessing emotional exhaustion was slightly changed to make the items more suitable for adolescents. Thus, instead of referring to coworkers and customers, we used a more open formulation, and referred to people in general (see Additional file 1 for items in German language).

#### Perceived stress

Perceived stress during the past month was assessed with the widely used 10-item Perceived Stress Scale (PSS) [63]. Participants were asked how often they find their lives to be overwhelming, uncontrollable, and unpredictable (e.g., “In the last month, how often have you felt that you were effectively coping with important changes that were occurring in your life?”, “In the last month, how often have you been upset because of something that happened unexpectedly?”). Answers were given on a 5-point Likert scale, ranging from 1 (never) to 5 (very often). Four items were reverse-poled and had to be recoded before calculating the sum score. Higher scores reflected higher subjectively perceived stress levels. Evidence for the reliability and validity of this instrument has been provided previously [64, 65]. In the present sample, the Cronbach’s alpha was satisfactory (α = .85).

#### Depressive symptoms

To assess depressive symptoms, all students filled in a German version of the Center for Epidemiologic Studies Depression Scale (CES-D), which is a self-report measure intended for research in general non-psychiatric populations. The instrument comprises 15 items assessing cognitive, emotional, motivational, behavioral, and somatic aspects associated with depression [66]. Previous studies with adolescents have demonstrated the validity and adequate internal consistency of the CES-D [67]. Items used a 4-point scale with response options of 0 (< 1 d/wk), 1 (1–2 d/wk), 2 (3–4 d/wk), and 3 (5–7 d/wk). The reverse-score was calculated for positively worded items, before building the sum score. Scores > 17 are generally considered as high [66]. The Cronbach’s alpha in the present sample was .86.

#### Life satisfaction

An overall judgement of participants’ satisfaction with life was obtained through the 5-item Satisfaction with Life Scale (SWLS) [68]. Answers were given on a Likert-scale from 1 (strongly disagree) to 7 (strongly agree). A sample item is: “In most ways my life is close to my ideal.” Validity and adequate reliability of this instrument have been reported previously [69, 70]. A sum score was built with higher scores reflecting higher satisfaction with life (the Cronbach’s alpha in the present sample was .84).

#### Statistical analyses

Correlational analyses were used to examine homogeneity and item-total correlations. Cronbach’s alpha coefficients were obtained to test internal consistency. We used confirmatory factor analysis (CFA) to examine factorial validity. We assumed that the 14 items would load on three different factors (six items on physical exhaustion, five items on cognitive weariness, three items on emotional exhaustion). Thus, the 3-factor CFA model was based on 14 observed measures and three latent constructs. Parameter estimation was conducted using maximum likelihood (ML), and multiple goodness-of-fit indexes were considered to examine how well the theoretical model fitted the empirical data [71]. Byrne [72] recommended that normed fit index (NFI) should be ≥ .95, comparative fit index (CFI) ≥ .95, Tucker Lewis Index (TLI) ≥ .95, and root mean square error of approximation (RMSEA) ≤ .05. According to Comrey and Lee [57], standardized factor loadings of ≥ .71 should be interpreted as excellent, ≥ .63 as very good, ≥ .55 as good, ≥ .45 as fair, and > .32 as poor. Correlations were employed to test convergent and discriminant validity, while we used univariate analyses of variance (ANOVA) to examine gender differences. To further test discriminant validity, we performed χ^2^ test to examine the overlap between students classified as having clinically relevant burnout symptoms and high depressive symptoms. CFA were performed with AMOS® 24 (IBM Corporation, Armonk NY, USA), all other analyses with SPSS® 24 (IBM Corporation, Armonk NY, USA).

### Results

As shown in Table 1, the SMBM overall score was 3.24 (*SD* = 0.99). Moreover, 12% (*n* = 30) of the participants were categorized as having clinically relevant levels of burnout (≥ 4.40). Compared to males, females had higher scores in physical exhaustion (females: *M* = 3.89, *SD* = 1.24, males: *M* = 3.19, *SD* = 1.03), cognitive weariness (females: *M* = 3.38, *SD* = 1.13, males: *M* = 2.93, *SD* = 1.07), and overall burnout (females: *M* = 3.44, *SD* = 0.99, males: *M* = 2.96, *SD* = 0.84). Compared to males (*n* = 2, 2.2%), females (*n* = 28, 17.5%) were also significantly overrepresented in the group with clinically relevant burnout symptoms, χ^2^(1, *N* = 247) = 12.45, *p* < .001.

**Table 1:** Descriptive statistics for the three samples, test of gender differences, and bivariate correlations between SMBM subscales and the overall SMBM index

	*M*	*SD*	Range	Skew	Kurt	ANOVA	
Sample 1: High school students (*N =* 249)							
Descriptive statistics						F	η^2^
Physical exhaustion	3.64	1.21	1.00–6.67	0.15	−0.51	20.35***	.076
Cognitive weariness	3.22	1.13	1.00–7.00	0.12	−0.15	9.58**	.037
Emotional exhaustion	2.34	1.57	1.00–6.67	1.04	1.24	0.34	.001
Overall SMBM index	3.24	0.99	1.00–5.93	0.04	−0.34	14.68***	.056
Bivariate correlations	1.	2.	3.	4.			
1. Physical exhaustion	–						
2. Cognitive weariness	.68***	–					
3. Emotional exhaustion	.35***	.39***	–				
4. Overall SMBM index	.91***	.85***	.42***	–			
Sample 2: Vocational students (*N* = 144)							
Descriptive statistics						F	η^2^
Physical exhaustion	3.15	1.09	1.17–5.83	0.29	−0.56	1.43	.010
Cognitive weariness	2.98	1.01	1.00–5.60	0.20	−0.73	0.04	.000
Emotional exhaustion	2.40	1.13	1.00–7.00	0.95	1.16	3.61	.025
Overall SMBM index	2.93	0.91	1.14–5.07	0.22	−0.78	1.05	.007
Bivariate correlations	1.	2.	3.	4.			
1. Physical exhaustion	–						
2. Cognitive weariness	.68***	–					
3. Emotional exhaustion	.48***	.52***	–				
4. Overall SMBM index	.91***	.88***	.72***	–			
Sample 3: Young elite athletes (*N* = 257)							
Descriptive statistics						F	η^2^
Physical exhaustion	3.55	1.13	1.33–6.67	0.30	−0.40	4.00*	.015
Cognitive weariness	3.35	1.08	1.00–7.00	0.43	0.34	5.18*	.020
Emotional exhaustion	2.80	1.16	1.00–6.67	0.53	0.01	0.19	.001
Overall SMBM index	3.32	0.96	1.29–6.79	0.46	0.33	2.91	.011
Bivariate correlations	1.	2.	3.	4.			
1. Physical exhaustion	–						
2. Cognitive weariness	.65***	–					
3. Emotional exhaustion	.49***	.47***	–				
4. Overall SMBM index	.89***	.86***	.71***	–			

Note. *Skew* = Skewness, *Kurt* = Kurtosis

*p < .05. **p < .01. ***p < .001

For each of the three SMBM subscales, the inter-item correlations exceeded the critical value of .20, and all item-total correlations were above the threshold of .40. The Cronbach’s alpha was .90 for physical exhaustion, .91 for cognitive weariness, .88 for emotional exhaustion, and .92 for the SMBM overall index.

**Fig. 1:**
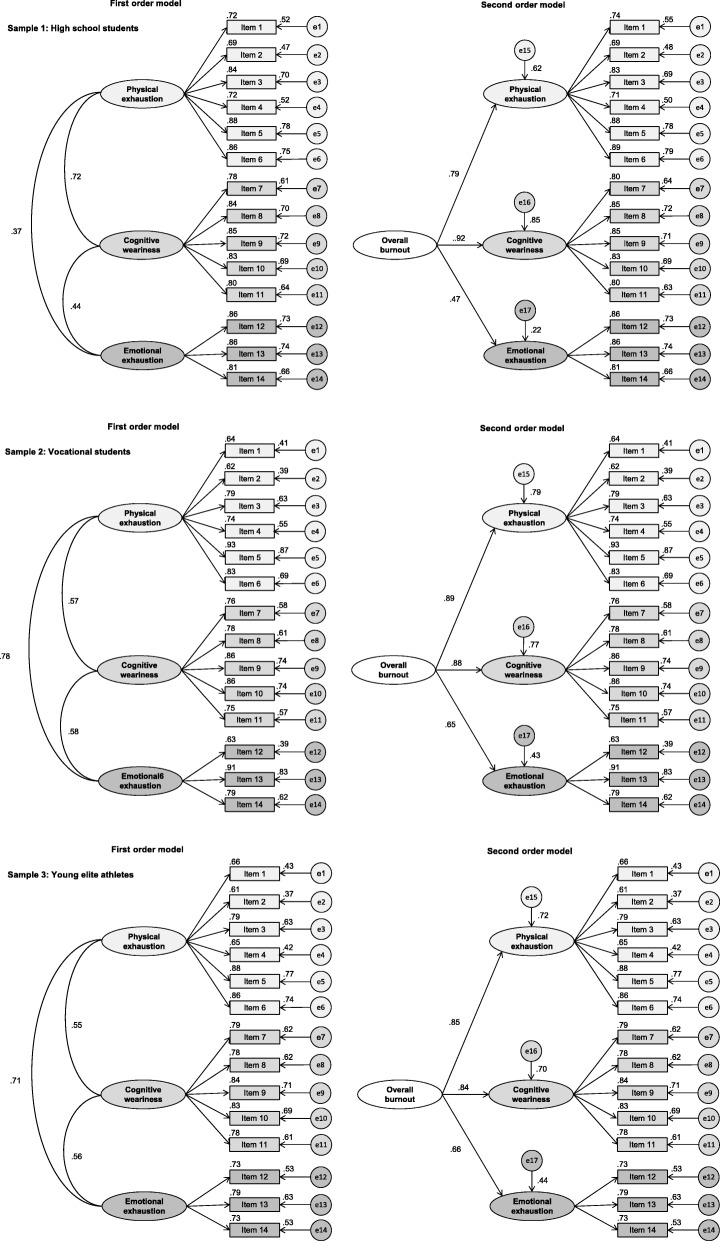
Factor loadings for confirmatory factor analysis for first- and second-order models, separately for high school students, vocational students and young elite athletes

The three-factor model fitted well with the empirical data in the CFA (Table 2). This conclusion applies for both the first- and second-order model. All factor loadings were very good to excellent (≥ .63). Figure 1 displays the measurement coefficients of the hypothesized three-factor models. In the first-order model, moderate-to-strong correlations were observed between the three SMBM dimensions (*r* = .37 to .72, *p* < .001).

**Table 2:** Goodness-of-fit indices

	First-order model				Second-order model			
	CFI	TLI	NFI	RMSEA	CFI	TLI	NFI	RMSEA
Sample 1: High school students								
Default model	.99	.99	.97	.03 (.00, .05)	.99	.99	.97	.03 (.00, .05)
Sample 2: Vocational students								
Default model	.96	.97	.93	.06 (.04, .08)	.96	.97	.93	.04 (.06, .08)
Sample 3: Young elite athletes								
Default model	.95	.96	.93	.06 (.05, .08)	.95	.96	.93	.06 (.05, .08)

*CFI* Comparative fit index, *TLI* Tucker Lewis index, *NFI* Normed fit index, *RMSEA* Root mean square error of approximation

With regard to convergent and discriminant validity, significant positive correlations were found between the SMBM and the PSS. While correlations with perceived stress were moderate to high for physical exhaustion, cognitive weariness, and overall burnout, only a weak (but significant) correlation occurred for emotional exhaustion. Significant negative correlations (of moderate magnitude) were found between satisfaction with life and three of four burnout indices (physical exhaustion, cognitive weariness, overall burnout). While the correlation of the emotional exhaustion subscale and life satisfaction pointed in the expected direction, the relationship was not significant. Moreover, moderate to strong correlations were found between the SMBM indices and depressive symptoms. Thus, the SMBM indices and the CES-D shared between 9 and 41% of variance. Finally, among those 28 students with high depressive symptoms (CES-D scores > 17), 53.6% (*n* = 15) also had clinically relevant burnout, whereas 46.4% did not (*n* = 13).

## Sample 2: Vocational students

### Methods

#### Participants and procedures

The sample consisted of 144 first-year students (age: *M* = 16.22 years, *SD* = 1.13, 97 males, 47 females) who were recruited from a vocational school in the Eastern part of Switzerland. Professions studied included polytechnicians, retail assistants, industrial clerks, structural draftsmen, and hair dressers. Questionnaires were completed within students’ classrooms under supervision of a trained research assistant (during the second month of the school year in September–October). Prior to data assessment, detailed information was given to the students about the purpose of the study and the voluntary nature of their participation. Students were assured confidentiality of their responses and informed written consent was sought from the participants.^2^ Inclusion criteria were: (a) informed written consent, (b) aged between 14 and 20 years, and (c) attending the selected vocational schools. None of the eligible participants were excluded. Ethical clearance was granted by the local ethical committee.

#### Burnout

To assess burnout symptoms, the participants filled in the 14-item Shirom-Melamed Burnout Measure (SMBM) [16], which has been described in detail in the introduction section (and above for sample 1). Additionally, vocational students completed the School Burnout Inventory (SBI) [27], which assesses *exhaustion at school* (4 items: e.g., “I feel overwhelmed by my schoolwork.”), *cynicism toward the meaning of school* (3 items: e.g., “I feel a loss of interest in my schoolwork.”), and *feeling a sense of inadequacy at school* (2 items: e.g., “I used to have higher expectations for my schoolwork than I have now.”). Answers on the SBI were given on a 6-point Likert scale ranging from 1 (completely disagree) to 6 (completely agree). Mean scores were built to obtain SBI subscale scores and an overall school burnout score, with higher values being indicative of higher school burnout levels. Evidence for the validity of this instrument has been reported previously [27]. In the present sample, the Cronbach’s alphas were .82 for the overall SBI index, .76 for exhaustion at school, .80 for cynicism toward the meaning of school, and .55 for sense of inadequacy at school.

#### Perceived stress

A 30-item version of the Adolescent Stress Questionnaire (ASQ) [73] was used to measure perceived general stress in this sample. Items pertain to ten domains of potential stressful experiences, including arguments at home, teachers expecting too much from them, pressure to fit in with peers or concerns about their future. For each stressor, answers were given on a 5-point Likert scale from 1 (not at all stressful or irrelevant) to 5 (very stressful). Satisfactory psychometric properties for this instrument have been reported previously [64, 74]. In the present sample, the Cronbach’s alpha was .90.

#### Depressive symptoms

Depressive symptoms were assessed with the 15-item CES-D [66], as previously described for sample 1 (Cronbach’s alpha in this sample was .81).

#### Life satisfaction

Satisfaction with life was assessed with the 5-item SWLS [68], as previously described for sample 1. In this sample, the Cronbach’s alpha was .82.

#### Statistical analyses

The same statistical procedures were used as with sample 1.

### Results

The SMBM overall mean score was 2.93 (*SD* = 0.91) (Table 1). In total, 6.9% (*n* = 10) of the participants reported clinically relevant burnout levels (SMBM scores ≥ 4.40). Similarly, 6.9% of the participants reported high levels of depressive symptoms (CES-D scores > 17). No significant gender differences existed among vocational students with regard to the SMBM overall index and subscale scores (Table 1). Boys (*n* = 7, 72.%) and girls (*n* = 3, 6.4%) were equally represented in the group with clinically relevant burnout symptoms, χ^2^(1, *N* = 144) = 0.03, *p* > .05.

For each of the three SMB*M* subscales, all inter-item correlations exceeded the critical value of .20. Similarly, all item-total correlations were satisfactory, with correlations exceeding the threshold of .40. The Cronbach’s alpha was .90 for physical exhaustion, .90 for cognitive weariness, .82 for emotional exhaustion, and .93 for the SMBM overall index.

With regard to factorial validity, the model fit of the three-factor model was adequate, with all goodness-of-fit indices being close or exceeding the targeted cut-offs, both for the first-order and second-order model (Table 2). Most of the factor loadings were very good or higher (≥ .63). Figure 1 provides the measurement coefficients of the hypothesized three-factor models. In the first-order model, relatively strong correlations were observed between the three SMBM dimensions (*r* = .57 to .78, *p* < .001).

With regard to convergent and discriminant validity (Table 3), the SMBM overall index was positively associated with participants’ perceived stress levels (*r* = .54, *p* < .001). Moderate negative correlations were observed between the SMBM indices and satisfaction with life (*r* = −.33 to −.41, *p* < .001), while moderate-to-strong correlations existed between the SMBM indices and the SBI overall scale (*r* = .41 to .69, *p* < .001). Additional analyses showed that participants with clinically relevant burnout symptoms (SMBM score ≥ 4.40) reported significantly higher overall school burnout levels (*M* = 3.62, *SD* = 0.54; *F*[1,247]=14.82 *p* < .001, η^2^ = .056) than peers below this threshold (*M* = 2.50, *SD* = 0.74). Moderate positive correlations were also found between the SMBM indices and the CES-D (*r* = .29 to .47, *p* < .001). Among those students with high levels of depressive symptoms (CES-D scores > 17), 80% (*n* = 8) did not have clinically relevant burnout levels (SMBM ≥ 4.40), whereas 20% (*n* = 2) did.

**Table 3:** Bivariate correlations between burnout symptoms, perceived stress, depressive symptoms and satisfaction with life

	Physical exhaustion	Cognitive weariness	Emotional exhaustion	Overall SMBM index
Sample 1: High school students (*N* = 249)				
Perceived stress (PSS)	.58***	.55***	.24***	.37***
Depressive symptoms (CES-D)	.64***	.60***	.30***	.51***
Satisfaction with life (SWLS)	−.39***	−.39***	−.10	−.25***
Sample 2: Vocational students (*N* = 144)				
Perceived stress (ASQ)	.35***	.50***	.42***	.54***
Depressive symptoms (CES-D)	.42***	.46***	.29***	.47***
Satisfaction with life (SWLS)	−.33***	−.34***	−.38***	−.41***
Overall school burnout (SBI)	.66***	.62***	.41***	.69***
Exhaustion at school	.47***	.51***	.29***	.52***
Cynicism toward school	.61***	.48***	.33***	.59***
Sense of inadequacy	.37***	.47***	.26***	.44***
Sample 3: Young elite athletes (*N* = 257)				
Perceived stress (PSS)	.48***	.48***	.37***	.55***
Depressive symptoms (PHQ9)	.38***	.37***	.23***	.39***
Satisfaction with life (SWLS)	−.36***	−.34***	−.35***	−.43***

*SMBM* Shirom-Melamed Burnout Measure, *PSS* Perceived Stress Scale, *CES-D* Center for Epidemiologic Studies Depression Scale, *PHQ9* Patient Health Questionnaire 9, *SBI* School Burnout Inventory. *SWLS* Satisfaction with Life Scale

*p < .05. **p < .01. ***p < .001

## Sample 3: Young elite athletes

### Methods

#### Participants and procedures

Sample 3 was composed of 257 young elite athletes attending Swiss Olympic Sport Classes in the North-Western, German-speaking part of Switzerland. Students from all Swiss Olympic Sport Classes from four Swiss cantons (Basel-City, Basel-Country, Argovia, Solothurn) were eligible for the study. Further inclusion criteria were: (a) informed written consent, (b) aged between 14 and 20 years. Students were assured confidentiality, and participation was voluntary. All students provided written informed consent before they responded to a written questionnaire, and none of the eligible participants were excluded.^2^ The data assessment took place during the second month of the school year (September–October). The study was approved by the local ethics committee. The sample consisted of 163 males and 94 females (age: *M* = 16.82 years, *SD* = 1.44).

#### Burnout

Burnout was assessed with the 14-item SMBM, in exactly the same way as with samples 1 and 2.

#### Perceived stress

Perceived stress was assessed with the 10-item PSS [63], as described above for sample 1 (Cronbach’s alpha in this sample was .80).

#### Depressive symptoms

Depressive symptoms were assessed with the 9-item Patient Health Questionnaire [75]. The PHQ-9 refers to the DSM-IV diagnosis criteria for major depressive disorder. Major depression is diagnosed if at least five of the nine depressive symptom criteria have been present on at least “more than half the days” in the past 2 weeks, and if one of the symptoms is depressed mood or anhedonia. The PHQ-9 can also be used to assess severity of depressive symptoms, with scores of > 14 reflecting moderately severe depression. The Cronbach’s alpha was .85 in the present sample.

#### Life satisfaction

Satisfaction with life was assessed with the 5-item SWLS [68], as previously described for samples 1 and 2. In this sample, the Cronbach’s alpha was .80.

#### Statistical analyses

The same statistical procedures were used as with samples 1 and 2.

### Results

As shown in Table 1, the SMBM overall score was 3.32 (*SD* = 0.96) in the total sample (Table 1). Furthermore, 12.1% (*n* = 31) of the participants reported clinically relevant burnout symptoms (≥ 4.4), whereas 8.9% (*n* = 23) reported moderately severe depression (PHQ-9 scores > 14). As in sample 1, females reported significantly higher scores (see Table 1) for physical exhaustion (females: *M* = 3.73, SD = 3.44, males: *M* = 3.44, *SD* = 1.10) and cognitive weariness (females: *M* = 3.55, SD = 1.13, males: *M* = 3.23, *SD* = 1.03). However, no significant gender differences were observed for the SMBM overall index and the emotional exhaustion subscale, and boys and girls were equally represented among participants with clinically relevant burnout levels.

For each of the three SMBM subscales, inter-item correlations were above .20, and all item-total correlations exceeded the threshold of .40 (for both males and females). The Cronbach’s alpha in the total sample was .88 for physical exhaustion, .90 for cognitive weariness, .80 for emotional exhaustion, and .92 for the SMBM overall index.

Good model fit was observed for the three-factor model in the CFA (Table 2). The model fit was comparable for the first- and second-order model. As shown in Fig. 1, most factor loadings were very good or excellent (≥ .63). In the first-order model, relatively strong correlations existed between the three SMB*M* subscales (*r* = .55 to .71, *p* < .001).

The significant positive correlations between the SMBM overall index and subscales and the PSS support the convergent validity of the instrument (Table 3). Significant negative correlations were observed between the four SMBM indices and satisfaction with life, while significant positive correlations of weak to moderate strength (*r* = .23 to .39, *p* < .01) were found between the SMBM indices and depressive symptoms. Finally, among those students with moderately severe depression (PHQ-9 scores > 14), 26.1% (*n* = 6) also had clinically relevant burnout levels, whereas 73.9% did not (*n* = 17).

## Testing measurement invariance of the SMBM across all samples

### Methods

#### Participants

To test measurement invariance across samples, the samples 1–3 were merged (*n* = 650). The overall sample consisted of 349 males and 301 females (age: *M* = 16.41 years, *SD* = 1.26).

#### Burnout

Across all samples, burnout symptoms were assessed in exactly the same way with the 14-item SMBM.

#### Statistical analyses

To test measurement invariance of the SMBM across all three samples, we carried out simultaneous multiple group comparisons. As reported previously, we assumed a 3-factor CFA model, based on 14 observed measures and three latent constructs. In this default model, all parameters were freely estimated. This model was then compared against a model in which all free factor loadings were set equally across the three samples. In a next step, we then tested this model against a model in which we assumed invariant factor loadings and inter-factor correlations (first-order model) or regression weights between the first- and second-order factors (second-order model). Again, multiple goodness-of-fit indices were considered to examine how well the theoretical model fitted with the empirical data (for more details, see description of statistical analyses for sample 1).

### Results

In the merged sample, the mean score was *M* = 3.40 (*SD* = 1.17) for physical exhaustion, *M* = 3.22 (*SD* = 1.09) for cognitive weariness, and *M* = 2.54 (*SD* = 1.17) for emotional exhaustion.

**Fig. 2:**
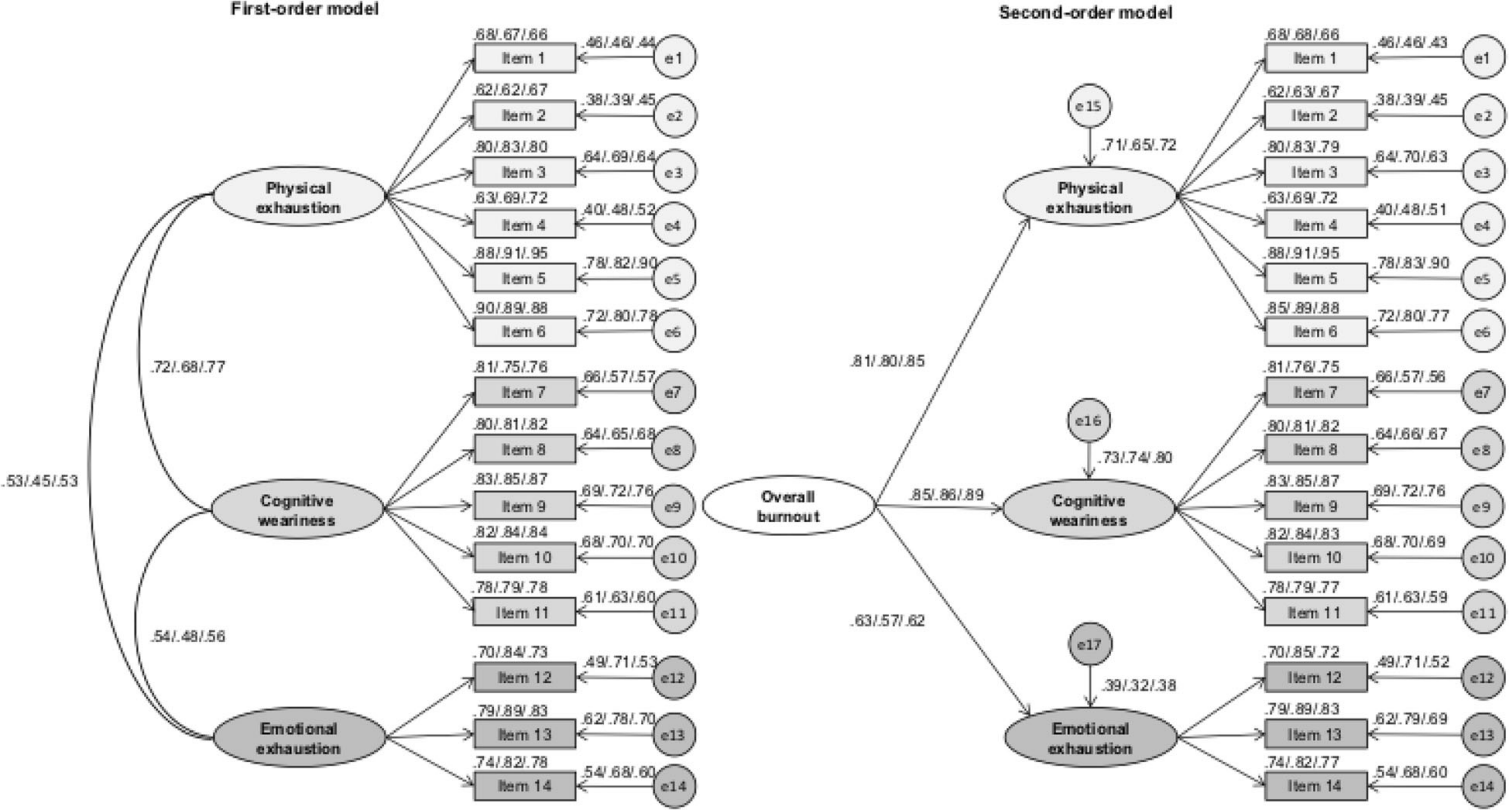
Factor loadings for confirmatory factor analysis for first- and second-order models, after having tested for measurement invariance across samples. First coefficient: young elite athletes. Second coefficient: high school students. Third coefficient: vocational students

The three-factor solution of the SMBM was supported (Fig. 2). As shown in Table 4, both the first-order and second-order model showed good fit between the theoretical model and the empirical data. The non-significant Δχ^2^-test showed that setting the factor loadings equal across samples did not lead to a worse model fit. Similarly, setting inter-factor correlations (first-order model) or regression weights (second-order model) equally across samples did not negatively impact the model fit (Δχ^2^-test: *p* = *ns*).

**Table 4:** Goodness-of-fit indices for testing measurement invariance across samples

	First-order model					Second-order model				
	CFI	TLI	NFI	RMSEA	Δχ^2^	CFI	TLI	NFI	RMSEA	Δχ^2^
Default model	.97	.96	.93	.04 (.03, .04)		.97	.96	.93	.04 (.03, .04)	
+ Factor loadings set equally across samples	.96	.96	.93	.04 (.03, .04)	*p* = .212	.96	.96	.93	.04 (.03, .04)	*p* = .212
+ Factor loadings and inter-factor correlations (first-order model) or regression weights (second-order model) set equally across samples	.96	.96	.93	.04 (.03, .04)	*p* = .363	.96	.96	.93	.04 (.03, .04)	*p* = .170

*CFI* Comparative fit index, *TLI* Tucker Lewis index, *NFI* Normed fit index, *RMSEA* Root mean square error of approximation

In the merged sample, the inter-item correlations exceeded the critical value of .20 for each of the three SMBM subscales, and all item-total correlations were above the threshold of .40. The Cronbach’s alpha was .90 for physical exhaustion, .91 for cognitive weariness, .88 for emotional exhaustion, and .92 for the SMBM overall index.

## Result and Discussion

The key finding of the present article is that among adolescents the SMBM has excellent psychometric properties and acceptable convergent/discriminant validity and can therefore be used as an alternative screening instrument in adolescent samples. Finally, our data confirm that clinically relevant burnout symptoms may already occur at young age.

In the introduction section, we have proposed six hypotheses, which we will now discuss in detail. First, we expected that the SMBM would have acceptable internal consistency across all samples [16, 55, 56]. This assumption was supported, with all Cronbach’s alpha coefficients exceeding the critical value of ≥ .70. Moreover, without exception, all inter-item correlations within the respective factor were ≥ .20, and all item-total correlations were ≥ .40. Based on the standards suggested by West et al. [76], we further found that the skewness (< 2) and kurtosis (< 7) of all SMBM indices were in the acceptable range.

Second, using CFA, evidence was found for the factorial validity of the SMBM across all our adolescent populations. Thus, our assumption that a three-factorial model would produce adequate fit was confirmed. In accord with prior research [55], almost all factor loadings were very good or excellent in our sample. The first-order model revealed moderate-to-strong correlations between the three latent factors, which is in line with previous research (cp. [6, 56, 62]). Shirom and Melamed [6] argued that energetic resources are individually possessed and expected to be closely interrelated, with deficits in one resource often leading to a deficit in other resource. Therefore, moderate-to-strong correlations between the latent factors were expected. Finally, in support of findings from data of Canadian workers [55], a second-order model produced equally good model fit, which corroborates the idea that the SMBM overall index can be used as a global/general measure of burnout.

Third, our analyses support the convergent validity of the SMBM across all samples. As reported in prior investigations with adult populations [58–60], our data suggest that the SMBM indices correlated at least moderately and positively with measures of self-perceived stress. Finally, across all samples moderate negative correlations were found for most of the SMBM indices and adolescents’ satisfaction with life, which is consistent with a previous study with Swiss vocational students [50].

Fourth, our findings support the discriminant validity of the SMBM. While our findings corroborate previous studies showing that the SMBM measures are at least moderately correlated with depressive symptoms [22, 24, 61], the strength of the correlations varied across our three study populations (*r* = .39 to .51), with the highest correlations found in high school students. While we acknowledge that there is a certain overlap between these two constructs, the fact that the SMBM overall index only shared between 15 and 26% of variance indicates that symptoms of burnout and depression are far from being identical constructs among adolescents. As suggested by Melamed et al. [9], some degree of overlap between burnout and depression is expected because both the definition of burnout and depression include fatigue and loss of energy as characterizing criteria. Schonfeld and Bianchi [22], however, recently argued that past research might have underestimated the overlap between burnout and depression. In their study with 1386 teachers, for instance, they reported a correlation of *r* = .77 (*p* < .001) between the SMBM and the PHQ-9. Moreover, 86% of the teachers identified as burned out met criteria for provisional diagnoses of depression. Nevertheless, this finding needs to be interpreted with caution because Schonfeld and Bianchi [22] used an arbitrary cut-off to classify participants into groups with versus without burnout (≥ 5.50), which was considerably higher than the empirically validated threshold suggested by Lundgren-Nilsson and colleagues [54]. We are aware that Lundgren-Nilsson et al.’s cut-off is based on the SMBQ (Shirom-Melamed Burnout Questionnaire = a slightly extended version of the SMBM) and therefore might not be perfectly applicable for the SMBM (despite a strong overlap between the items of the two instruments). Nevertheless, we still prefer this empirically derived threshold in comparison to any arbitrary cut-off value. In summary, we believe that our findings corroborate the discriminant validity of the SMBM because the percentage of adolescents who were simultaneously classified into the group with high burnout levels and high levels of depressive symptoms was not too high, with 20% among vocational students, 26% among young elite athletes, and 53% among high school students.

Fifth, our assumption that female participants would score higher on burnout symptoms than male participants was partly confirmed. While significant gender differences were found in high school students and young elite athletes, boys and girls did not differ among vocational students. The inconsistent pattern of finding is in line with previous research. Thus, while studies with adolescents mostly supported gender differences [27, 45], among adult workers, male and female participants did not always differ from each other [59, 62]. In the present study, it is possible that the male and female vocational students did not differ from each other because they generally reported lower burnout symptoms than high school students or young elite athletes. Accordingly, floor effects might have decreased the odds for higher variances and for detecting a higher variability in the measurements. The fact that vocational students had the lowest SMBM scores was a surprise because it has been suggested that the vocational students are exposed to workloads similar to adult workers and are therefore at risk for elevated stress levels [51]. However, the vocational students participating in the present study were in their first year. Thus, it is likely that burnout symptoms will increase in the third or fourth year of vocational education and training. Finally, in line with previous studies [55, 62], physical fatigue turned out to be the most affected burnout dimension across all three samples, followed by cognitive weariness and emotional exhaustion.

Sixth, our data supported the notion that the measurement model is invariant across samples. A multiple group comparison showed that the overall model fit is excellent. Setting factor loadings and inter-factor correlations/regression weights equal across samples did not have a negative impact on the goodness-of-fit indices. Thus, we claim that the SMBM seems to perform equally well in various adolescent samples.

Despite the novelty of our findings, several limitations should be taken into consideration: For instance, due to the cross-sectional nature of our data, it was not possible to test predictive validity and test-retest reliability. Moreover, all three samples consisted of non-clinical populations and we only used measures of self-reported depressive symptoms to examine discriminant validity. Thus, without formal clinical diagnoses, we were not able to validate the cut-off for clinically relevant burnout (≥ 4.4), which was previously established by Lundgren-Nilsson et al. [54].

Implications for clinical practice are that 7–12% of adolescents reported symptoms pointing towards clinically relevant levels of burnout. This percentage is comparable to adult populations [23, 59] and shows that more systematic efforts are needed to prevent burnout symptoms. As known from adult studies, burnout symptoms have a relatively high temporal stability, and may track across extended periods of time [77, 78]. In future research, researchers could test strategies to empower students to maintain a better stress-recovery balance [79].

## Conclusions

The SMBM is a frequently used tool in international burnout research. Our study reveals that this instrument has adequate psychometric properties and satisfactory convergent and factorial validity among adolescents. Hence, the SMBM can be used among young people to gather information for screening and treatment planning, which may be of particular interest during the transition from school to working life. There is a clear need to validate the cut-off score for clinically relevant burnout, in order to more accurately judge the clinical significance of the symptoms in early screening.

## Additional file

Additional file 1: Shirom-Melamed Burnout Measure – German Adolescent Version. (DOCX 17 kb)

## Abbreviations

ASQ: Adolescent Stress Questionnaire
BBI-15: Bergen Burnout Indicator 15
CES-D: Center for Epidemiologic Studies Depression Scale
CFA: Confirmatory Factor Analysis
CFI: Comparative Fit Index
DSM-IV: Diagnostic and Statistical Manual of Mental Disorders (4th Edition)
MBI: Maslach Burnout Inventory
ML: Maximum likelihood
NFI: Normed Fit Index
PHQ-9: 9-item Patient Health Questionnaire
PSS: Perceived Stress Scale
RMSEA: Root Mean Square Error of Approximation
SBI: School Burnout Inventory
SMBM: Shirom-Melamed Burnout Measure
SWLS: Satisfaction with Life Scale
TLI: Tucker Lewis Index

## Footnotes

1 Data with regard to the SBI was only available in sample 2 (vocational students). Thus, as suggested by Hypothesis 3, convergent validity between the SMBM and the SBI was only tested in one sample.

2 In the case of studies with minor participants, the Swiss national legislation (Humanforschungsgesetz HFG) makes a distinction between children (≤ 14 years) and adolescents (15–18 years). In studies with children, parental informed written consent is needed (Art. 22). In studies with adolescents, informed written consent of the adolescent is required. Moreover, parental informed written consent is needed for studies, which bear more than minimal risks. However, all our studies were classified in the lowest risk category. Therefore, no parental informed written consent was required.
