# Supplementary material for: Psychometric properties of the Shirom-Melamed Burnout Measure (SMBM) among adolescents: results from three cross-sectional studies
Source: BMC Psychiatry. 2018 Aug 25;18:266. doi: 10.1186/s12888-018-1841-5 (PMC6109266; doi:10.1186/s12888-018-1841-5)
Supplement: Supplementary file 1 — Shirom-Melamed Burnout Measure – German Adolescent Version. (DOCX 17 kb) [file 12888_2018_1841_MOESM1_ESM.docx]

**Additional file 1 Shirom-Melamed Burnout Measure – German Adolescent Version**

| Wenn Du an den letzten Monat denkst, wie hast Du Dich dann gefühlt?  *(English: Please indicate how often, in the past 30 days, you have felt each of the following feelings.)* | Nie/fast nie  *(never/*  *almost never)* | Sehr selten  *(very infre-quently)* | Ziemlich selten  (quite infre-quently) | Manch-mal  *(some-times)* | Ziem-lich oft  *(quite fre-quently)* | Sehr oft  *(very fre-quently)* | Immer/  fast immer *(always/almost always)* |
| --- | --- | --- | --- | --- | --- | --- | --- |
|  |  |  |  |  |  |  |  |
| 1. Ich fühlte mich müde. *(English: I feel tired.)* |  |  |  |  |  |  |  |
| 2. Ich hatte keine Energie, um morgens zur Schule zu gehen. *(English: I have no energy for going to school in the morning.)* |  |  |  |  |  |  |  |
| 3. Ich fühlte mich körperlich völlig ausgelaugt. *(English: I feel physically drained.)* |  |  |  |  |  |  |  |
| 4. Ich hatte die Nase voll. *(English: I feel fed up.)* |  |  |  |  |  |  |  |
| 5. Ich hatte das Gefühl, dass meine Batterien leer sind. *(English: I feel like my „batteries“ are „dead“.)* |  |  |  |  |  |  |  |
| 6. Ich fühlte mich ausgebrannt. *(English: I feel burned out.)* |  |  |  |  |  |  |  |
| 7. Mein Denken war verlangsamt. *(English: My thinking process is slow.)* |  |  |  |  |  |  |  |
| 8. Ich hatte Schwierigkeiten, mich zu konzentrieren. *(English: I have difficulty concentrating.)* |  |  |  |  |  |  |  |
| 9. Ich hatte das Gefühl, nicht klar zu denken. *(English: I feel I am not thinking clearly.)* |  |  |  |  |  |  |  |
| 10. Ich hatte das Gefühl, beim Denken nicht bei der Sache zu sein. *(English: I feel I am not focused in my thinking.)* |  |  |  |  |  |  |  |
| 11. Ich hatte Schwierigkeiten, über komplexe Dinge nachzudenken. *(English: I have difficulty thinking about complex things.)* |  |  |  |  |  |  |  |
| 12. Ich fühlte mich nicht in der Lage, mich auf die Bedürfnisse von anderen Menschen einzustellen. *(English: I feel I am unable to be sensitive to the needs of other people.)* |  |  |  |  |  |  |  |
| 13. Ich fühlte mich nicht in der Lage, emotional auf andere Menschen einzugehen. *(English: I feel I am not capable of investing emotionally in other people.)* |  |  |  |  |  |  |  |
| 14. Ich fühlte mich nicht in der Lage, mich in andere Menschen hineinzuversetzen. *(English: I feel I am not capable of being sympathetic to other people.)* |  |  |  |  |  |  |  |
